# Independent components of wing kinematics in the fruit fly *Drosophila*

**DOI:** 10.1186/1471-2202-14-S1-P429

**Published:** 2013-07-08

**Authors:** Soma Chakraborty, Jan Bartussek, Steven N Fry, Martin Zapotocky

**Affiliations:** 1 Institute of Physiology, Academy of Sciences of the Czech Republic, Prague, 14220, Czech Republic; 2 First Faculty of Medicine, Charles University, Prague, 12108, Czech Republic; 3 Institute of Neuroinformatics, University of Zurich and ETH Zurich, Zurich, CH-8057, Switzerland; 4 SciTrackS GmbH, CH-8118 Pfaffhausen, Switzerland

## 

Flies are known for their supreme flight maneuverability that is facilitated by their highly specialized flight apparatus. While large power muscles generate the basic wingbeat, flies control their flight with about 6 miniscule steering muscles per wing side. It is thought that a small number of steering muscle co-activation synergies give rise to the fast and precise changes in wingbeat responsible for flight maneuvers [1]. Complementary to existing electrophysiological studies of selected muscles, we developed a statistical method based on independent component analysis [2] to classify the wing stroke patterns of the fruit fly. Our method identifies components of the wing motion that are maximally statistically independent of each other; such components may be viewed as manifestations of basic neuromotor flight control modes resulting from specific muscle synergies.

We recorded the wing motion of tethered flying fruit flies using a high speed computer vision system together with a novel procedure for online extraction of wing position (TrackFast, SciTrackS.com) [3]. This setup enables a continuous recording of 10 000 wingbeat cycles at a rate of 6000 Hz (~16 data points / cycle); such long duration recordings permit statistical analysis. We defined stroke angles at 8 distinct phases of the wingbeat cycle as separate time series, and thus each test flight was represented by a set of 16 signals (both wings). For the ICA analysis we used the MILCA algorithm [1] and judged its success by estimating pairwise mutual information of the least dependent components (LDCs) obtained.

Based on the power spectral densities of the obtained LDCs, we separated random changes in wing trajectory from variations reflecting possible flight maneuvers. Among the components with significant temporal structure, we identified 7 distinct types of LDCs, of which three are related to well-known flight maneuvers. To interpret the corresponding stroke modifications, we reconstructed the stroke trajectories by inverse transforming LDCs such that the variance of all but that of the one of interest is suppressed.

**Figure 1:**
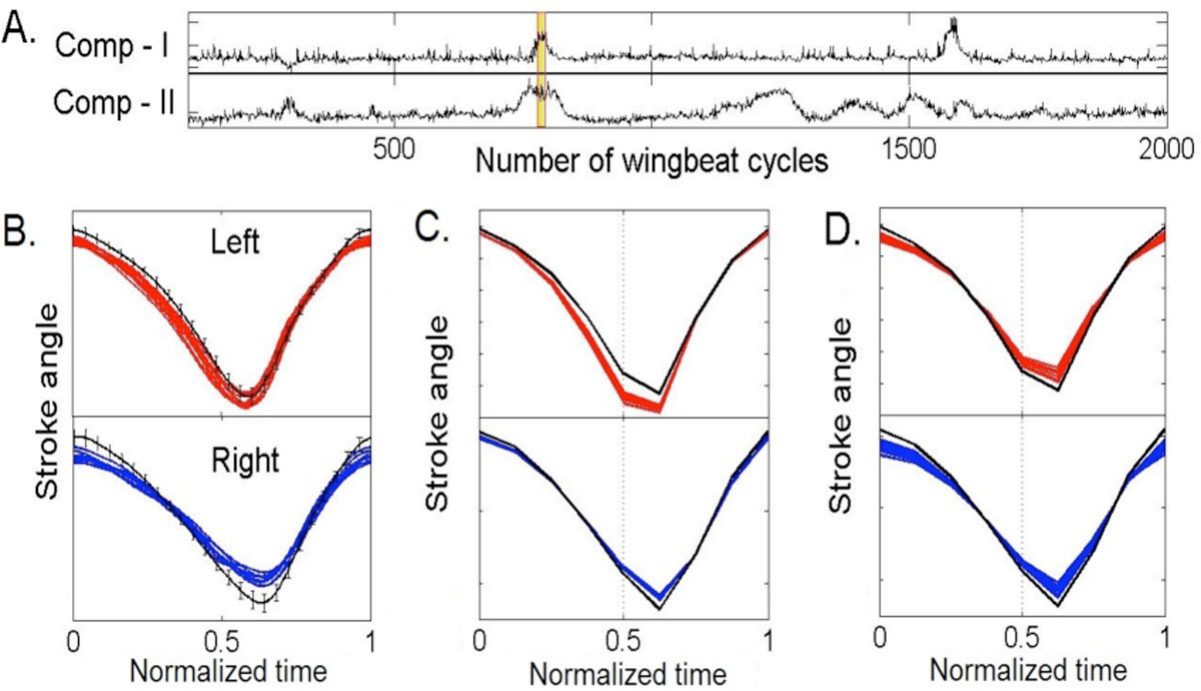
**A) Time course of two LDCs from a flight segment of 2000 wingbeat cycles**. **B) **Recorded stroke trajectories of 10 cycles (highlighted in A). Red: left wing; blue: right wing. Black line shows mean trajectory during the whole flight segment. **C, D) **Reconstructed stroke trajectories of the same 10 cycles with variances of all components but Comp-I (in C) or Comp-II (in D) suppressed. The left-right antisymmetric variation in C corresponds aerodynamically to a yaw saccade maneuver, and the symmetric variation in D to a lift maneuver.

In some instances our MILCA analysis resolved complex trajectory variations into simpler events characteristic of LDCs (Figure 1). This implies that muscle synergies separated in individual LDCs linearly superpose when acting simultaneously. In conclusion, our findings suggest that a limited number of independent control modes are active during a given flight test and that the muscle synergies corresponding to these modes operate nearly independently from each other.
